# Water activated disposable paper battery

**DOI:** 10.1038/s41598-022-15900-5

**Published:** 2022-07-28

**Authors:** Alexandre Poulin, Xavier Aeby, Gustav Nyström

**Affiliations:** 1 Laboratory for Cellulose and Wood Materials, Swiss Federal Laboratories for Materials Science and Technology (Empa), 8600 Dübendorf, Switzerland; 2 Department of Health Sciences and Technology, ETH Zürich, 8092 Zürich, Switzerland

**Keywords:** Electronic devices, Electrical and electronic engineering

## Abstract

We developed a disposable paper battery aiming to reduce the environmental impact of single-use electronics for applications such as point of care diagnosis, smart packaging and environmental sensing. The battery uses Zinc as a biodegradable metal anode, graphite as a nontoxic cathode material and paper as a biodegradable substrate. To facilitate additive manufacturing, we developed electrodes and current collector inks that can be stencil printed on paper to create water-activated batteries of arbitrary shape and size. The battery remains inactive until water is provided and absorbed by the paper substrate, taking advantage of its natural wicking behavior. Once activated, a single cell provides an open circuit potential of 1.2 V and a peak power density of 150 µW/cm^2^ at 0.5 mA. As a proof of concept, we fabricated a two cell battery and used it to power an alarm clock and its liquid crystal display.

## Introduction

Over the last decades, we have witnessed an ever-increasing use of electronic devices, leading in turn to electronic waste (e-waste) becoming the world's fastest growing waste stream^1,2^. Mitigating the associated environmental risks requires advances at the material and device levels, for instance by moving towards more environmentally friendly materials and improving the resource recovery rate. There has been notable progress in this direction, for example with the development of green power supply technologies such as biodegradable photovoltaics^3,4^, energy harvesters^5,6^ and supercapacitors^7,8^. However, there is still limited research on biodegradable primary batteries, a complementary and versatile source of energy that can provide higher energy density and more stable operation.

Battery research predominantly focuses on performance^9,10^, constantly progressing towards higher energy and power densities, faster charging rates and improved operation stability. This is mainly achieved by developing new materials tailored to the requirements of lithium-ion cells that currently dominate the market^11^. However, with a rising awareness of the e-waste problem and the emergence of single-use electronics for applications like environmental sensing and food monitoring, there is a growing need for low environmental impact batteries. This shift from traditional performance-oriented figure of merits creates new opportunities for unconventional materials and designs that can provide a balance between performance and environmental impact.

Aqueous primary batteries based on inorganic materials such as magnesium (Mg), iron (Fe), tungsten (W) and molybdenum (Mo) have recently emerged as promising candidates for use in high-energy density transient batteries^12,13^. Organic alternatives have also been demonstrated using, for instance, naturally occurring melanin and quinone in a biodegradable aqueous redox flow battery^14^. Although promising advances have been made in recent years, additive manufacturing of biodegradable batteries remains an important scientific challenge^15^.

Cellulose, in the form of paper, has an undeniable historical importance. Following its millennial use as a carrier substrate for information and knowledge transfer, it has over the last decade experienced renewed interest as an advanced material in a wide range of applications including in biomedical diagnostics^16^, as information display^17^ and for energy storage^8,18^. Batteries and supercapacitors have also been developed using cellulose as a high surface area template for redox active materials, or as a low-cost substrate by coating functional dispersions onto pre-formed paper or foam^19–22^. However, several unique properties of cellulose such as its intrinsic biodegradability, hygroscopic nature and wicking behavior have so far been poorly utilized.

In this work, we present a printed paper battery developed to power single-use disposable electronics and to minimize their environmental impact. The battery is based on a metal-air electrochemical cell that uses Zinc as a biodegradable metal in the anode, graphite in the cathode, paper as a separator between the electrodes, and a water-based electrolyte. In addition to paper’s inherent biodegradability, sustainability and low cost, this design takes advantage of its natural wicking behavior and hygroscopic nature; The battery remains inactive until it contacts with water which then passively absorbs and transports across the paper membrane, thus activating the battery. The anode and cathode materials developed in this work are compatible with additive manufacturing techniques and we demonstrate that the battery can be stencil printed in a wide range of shapes and sizes.

The design of the battery, materials formulation, fabrication process, and characterization protocols are described in the “Methods” section. The materials requirements, ink rheological properties, and performance of the battery are presented in the “Results and discussion” section.

## Methods

### Battery design

**Figure 1:**
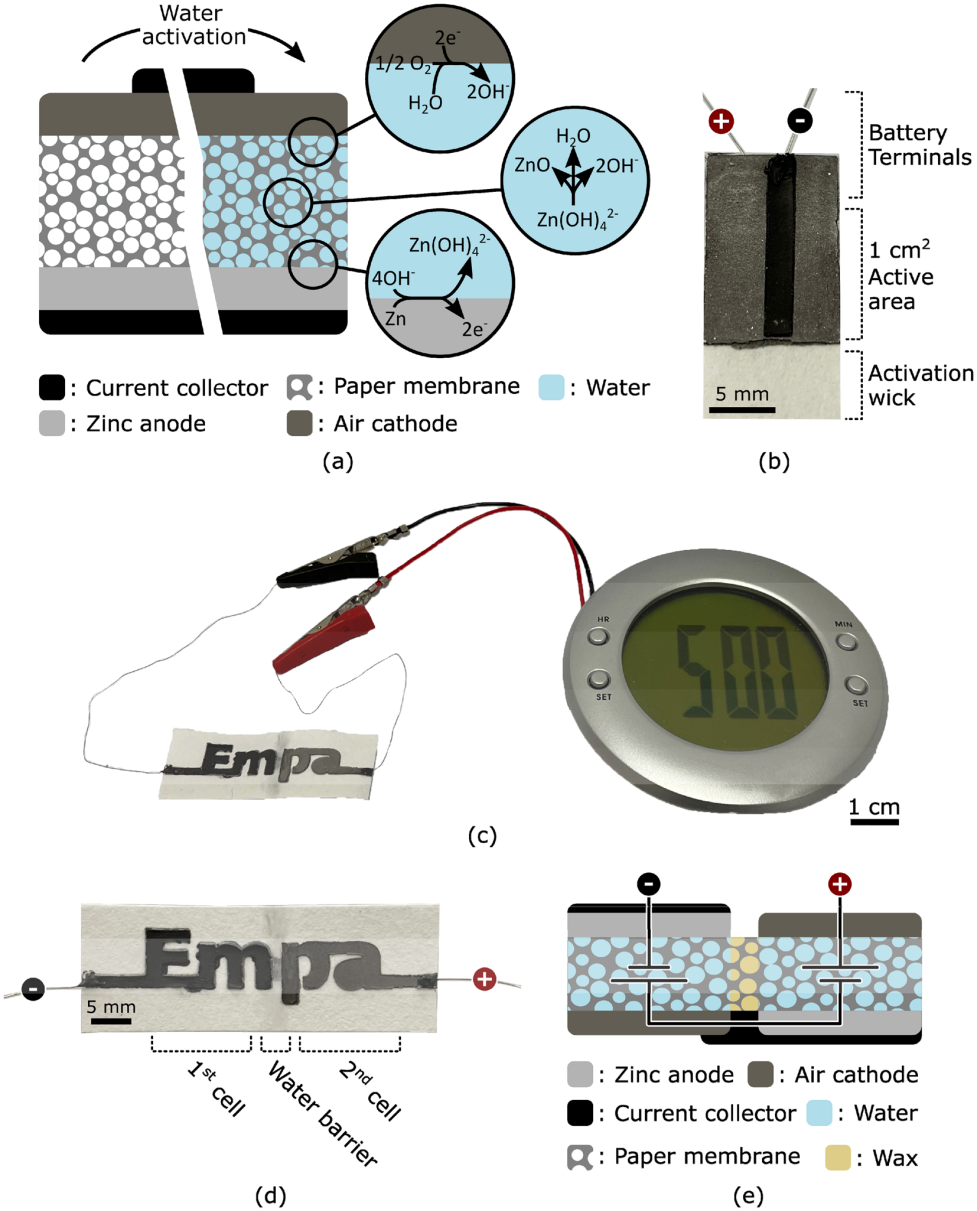
(**a**) Illustration of the water-activated paper battery. Its electrochemical (EC) cell is composed of a paper membrane sandwiched between a zinc-based cathode and a graphite-based air cathode. Carbon-based current collectors are used to extract charges from the EC cell and contact with external circuitry. The device remains inactive until water, which serves as the electrolyte, is provided to the system and permeates the paper membrane. (**b**) Picture of single cell battery fabricated by stencil printing on filter paper. The device is activated by immersing the wick in water or any aqueous solution. At the battery terminals, the filter paper is impregnated with wax to avoid electrochemical reactions of the lead wires and to provide mechanical stability. (**c**) Photograph of a stencil-printed paper battery with a design that spells the name of our research institution (Empa). The battery can run low-power electronics like the liquid crystal display (LCD) alarm clock shown in this photograph. The device is composed of two electrochemical cells that are separated by a water barrier as depicted in the (**d**) photograph, and connected in series as illustrated in the (**e**) schematic cross-section of the battery with its overlaid equivalent circuit (for ideal voltage sources).

The single cell battery is composed of a paper substrate sandwiched between the air cathode and a current collector on one side, and the zinc anode and a current collector on the opposite side. Figure 1a presents a schematic cross-section of the device and illustrates its water activation process. The battery is manufactured without electrolyte, effectively maintaining the anode and cathode isolated from one another. When water is provided to the system, it readily absorbs and diffuses through the paper substrate, thus dissolving NaCl dispersed in the paper and thereby activating the electrochemical cell. During discharging, the zinc anode is oxidized while an oxygen reduction reaction occurs at the cathode. Because the cathode reaction uses oxygen from ambient air, the airtight current collector located on this side of the device is limited in size. This design maximizes oxygen flow while maintaining the contact resistance as low as possible. Figure 1b presents a picture of the fabricated battery where the cathode appears in gray, the current collector in black and the substrate in white. The paper substrate extends beyond the 1 cm^2^ active area to create an activation wick where water can absorb into the system. Contrarily, the substrate is made hydrophobic on the terminals end to avoid undesired electrochemical reactions with the connecting wires. This single cell battery provided a 1.2 V open circuit potential.

Multiple electrochemical cells can be printed on the same substrate and connected in series to achieve higher open circuit potentials. Figure 1c shows a two cell battery with a 2.4 V open circuit potential powering an alarm clock and its liquid crystal display. As a demonstration of the design flexibility provided by our approach, the battery spells the name of our research institution (Empa). A closer view is presented in Fig. 1d where the anode of the left cell appears in black and the cathode of the right cell in appears in grey. The cells are separated by a hydrophobic region as illustrated in the schematic cross-section presented in Fig. 1e, overlaid with the equivalent circuit (for ideal voltage sources).

### Ink preparation and characterization

The cathode ink is composed of 15 wt% shellac (Shellac Orange by Kremer Pigment, Germany), 30 wt% ethanol, 47 wt% graphite flakes (7–10 µm flakes by Alfa Aesar, USA) and 8 wt% polyethylene glycol (PEG 400 by VWR, Switzerland). Shellac is dissolved in ethanol, after which graphite and PEG are added. The resulting blend is mixed in a planetary mixer (DAC600 by Hauschild SpeedMixer, Germany) for 1 min at 2300 rpm.

The anode ink is composed of 2.5 wt% shellac (Shellac Orange by Kremer Pigment, Germany), 5.5 wt% ethanol, 89.5 wt% zinc powder (Zinc fine powder by VWR Chemicals, Switzerland) and 2.5 wt% polyethylene glycol (PEG 400 by VWR, Switzerland). Shellac is dissolved in ethanol, after which zinc powder and PEG are added. The resulting blend is mixed in a planetary mixer (DAC600 by Hauschild SpeedMixer, Germany) for 1 min at 2300 rpm.

The current collector ink is composed of 21.5 wt% shellac (Shellac Orange by Kremer Pigment, Germany), 41.5 wt% ethanol, 6.5 wt% carbon black (Carbon ECP by Lion Specialty Chemicals Co., Ltd Japan), 26.5 wt% graphite flakes (7–10 µm flakes by Alfa Aesar, USA) and 4 wt% polyethylene glycol (PEG 400 by VWR Chemicals, Switzerland). Shellac is dissolved in ethanol, after which carbon black, graphite and PEG are added. The resulting blend is mixed in a planetary mixer (DAC600 by Hauschild SpeedMixer, Germany) for 1 min at 2300 rpm, and ball milled for 10 min at 800 rpm (Pulverisette 7 by Fritsch, Germany).

The wax oleogel is composed of 50 wt% carnauba wax (Carnauba wax flakes by Thermo Scientific, Switzerland) and 50 wt% rapeseed oil (Rapeseed Oil Research only by MP biomedicals, USA). The oil and wax are combined in a metal dish which is then placed on a hot plate, heated above the melting temperature of carnauba wax and thoroughly stirred. The resulting blend is removed from heat and cooled down to room temperature.

The rheology of the anode, cathode and current collector was characterized on a rotational and oscillatory rheometer (MCR301 by Anton Paar, Austria) using a plate-plate geometry with a 1 mm gap. The measurements were made at 20 °C using a Peltier hood to ensure uniform temperature across the sample and minimized solvent evaporation. Viscosity was measured as a function of shear rate using rotational test at controlled shear rate. Storage and loss modulus were measured as function of stress using oscillatory test at controlled strain.

### Fabrication protocol

The fabrication process of the single cell battery shown in Fig. 1b is illustrated in Figure S1 of the Supplementary Information and briefly summarized here. The process starts with a paper substrate (paper filter grades 597 by Whatman Product, United Kingdom) cut to a slightly larger size than the final desired battery. One end of the substrate is immersed in melted wax to create a hydrophobic region where the battery terminals will be located. The substrate is then immersed in a 3 M aqueous solution of NaCl serving as the ionic species for the electrolyte and dried. The cathode is stencil-printed and dried at 60 °C for 10 min. The same printing technique and drying conditions are used to subsequently pattern the top current collector, anode and bottom current collector. The sample is cut to its final shape and lead wires are connected (using the current collector ink) to create the battery terminals.

The fabrication process of the two cell battery shown in Fig. 1c is illustrated in Figure S2 of the Supplementary Information and briefly summarized here. The process starts with a paper substrate cut to a slightly larger size than the battery design. A stripe of wax oleogel is stencil-printed to create two regions separated by a hydrophobic barrier. The substrate is then placed on a 100 °C hotplate for a few seconds to let the wax melt and infuse the paper. The following steps are essentially the same as for the single cell battery.

### Electrochemical measurements

The performance of the battery was characterized using an electrochemical workstation (Model 600E by CH Instruments, Inc., USA). The open circuit potential was measured as a function of time using the Open Circuit Potential – Time (OCPT) settings. The battery was activated during this measurement in order to measure the activation time. The frequency response was measured using the Impedance spectroscopy (AC IMP) settings, scanning from 1 Hz to 1 MHz with an amplitude of 0.02 V at an initial voltage equal to the open circuit potential. The power capability was measured using the multi-current steps (ISTEP) settings, measuring the operating voltage for currents ranging from 0.1 mA to 1 mA with 0.1 mA current steps. The power was then calculated as the operating voltage times the current. The discharge curve was measured using the Chronopotentiomety (CP) settings, drawing 0.1 mA from the battery and recording the operating voltage as a function of time.

## Results and discussion

### Battery materials requirements

**Figure 2:**
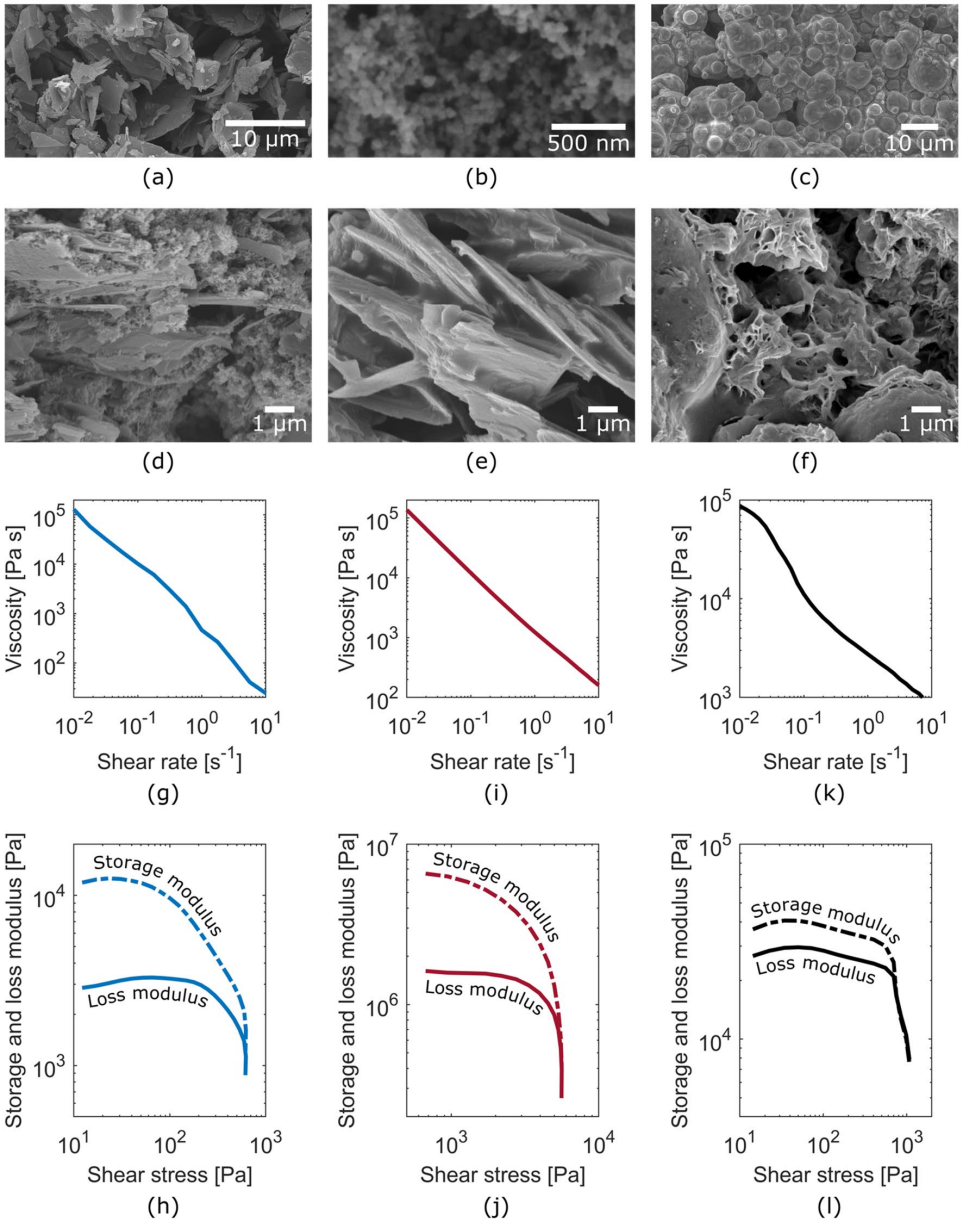
SEM micrographs of the (**a**) graphite flakes, (**b**) carbon black and (**c**) zinc particles comprised in the battery. (**d**) SEM micrograph of the current collector showing the densely packed graphite flakes, carbon black and shellac composite structure. (**e**) SEM micrograph of the cathode showing the loosely packed graphite flakes and shellac composite structure. (**f**) SEM micrograph of the anode on a partially discharged battery showing the porous shellac matrix that remains after zinc is consumed. (**g**) Graph of the viscosity as a function of shear rate showing the shear-thinning behavior of the current collector ink. (**h**) Graph of the storage and loss modulus as a function of shear stress showing the 600 Pa yield stress of the current collector ink. (**i**) Graph of the viscosity as a function of shear rate showing the shear-thinning behavior of the cathode ink. (**j**) Graph of the storage and loss modulus as a function of shear stress showing the 5600 Pa yield stress of the cathode ink. (**k**) Graph of the viscosity as a function of shear rate showing the shear-thinning behavior of the anode ink. (**l**) Graph of the storage and loss modulus as a function of shear stress showing the 750 Pa yield stress of the anode ink.

The role of the current collector is to connect the cathode and anode to external circuitry. The ideal material therefore provides high electrical conductivity and low contact resistance. It is also important that the current collector doesn’t chemically react or dissolve in contact with the electrolyte. While metals such as aluminum and copper are typically used as current collector, their use for disposable electronics is a waste of valuable resources. Our current collector ink is composed of graphite flakes, carbon black, shellac and ethanol^23^. The graphite flakes (Fig. 2a) and carbon black (Fig. 2b) provide electrical conductivity to the composite (Fig. 2d). The smaller carbon black particles improve electrical contact between the larger and more conductive graphite flakes.

The role of the air cathode is to support the oxygen reduction reaction (ORR). The ideal material therefore maximizes the density of reaction sites and allows for oxygen in ambient air to reach these sites. Transition metals like Platinum or Cobalt are typically used as catalyst for the ORR reaction, effectively reducing the activation energy and increasing the reaction rate. We avoided this type of additive for our disposable battery because of the environmental concerns associated with the bioaccumulation of heavy metals. Our cathode ink is composed of graphite flakes, shellac and ethanol. Once dried, the graphite flakes (Fig. 2a) and the shellac create a porous structure (Fig. 2e). Compared to the current collector, the cathode provides a higher density of reaction sites and is permeable to air which allows for oxygen from ambient air to reach the reaction sites. It however has a lower electrical conductivity.

The role of the anode is to support the zinc oxidation reaction. In most commercial zinc air batteries, the anode is a piece of pure zinc which also serves as the current collector and as a structural part of the battery’s outer shell. This configuration simplifies fabrication and assembly, but a large proportion of the available zinc remains unused. While this is a viable approach for a closed loop system where devices are recycled at the end of their useful life, it’s not compatible with disposable applications. Our anode ink is composed of zinc powder, shellac and ethanol. The zinc (Fig. 2c) particles embedded in the shellac matrix are consumed during discharging, leaving behind a porous matrix of shellac (Fig. 2f). This approach provides the design flexibility of additive manufacturing and uses zinc discerningly i.e. only as the reactive element in the anode. The structural integrity is provided by the paper substrate and connections to outside circuitry are achieved with our carbon-based current collector.

### Ink rheological properties

We characterized the rheological properties of our inks by measuring their shear thinning behavior and yield stress, two important properties for additive manufacturing technique such as robocasting, screen printing and stencil printing. The current collector ink exhibits shear thinning behavior and a yield stress of 600 Pa as shown in Fig. 2g and h, respectively. While only stencil printing is investigated in this work, we recently demonstrated the compatibility of this current collector ink with robocasting and screen printing. The cathode ink exhibits shear thinning behavior and a yield stress of 5600 Pa as shown in Fig. 2i and j, respectively. This rheology worked well for stencil printing. The yield stress is, however, at the higher end of the range for extrusion-based techniques like robocasting. For this type of additive manufacturing, the ink formulation could be slightly modified in order to achieve a yield stress closer to 10^3^ Pa. The anode ink exhibits shear thinning behavior and a yield stress of 750 Pa as shown in Fig. 2k and l, respectively. The loss tangent (i.e. storage to loss modulus ratio) is lower than for the other inks, indicating a weaker gel. This had no impact on this work and this rheology worked well for stencil printing. A weaker gel, however, ultimately defines the maximum aspect ratio above which the gel starts flowing under its own weight.

### Battery performance

The performance of our paper battery was characterized using the device shown in Fig. 1b, and all measurements were made on the same device.

**Figure 3:**
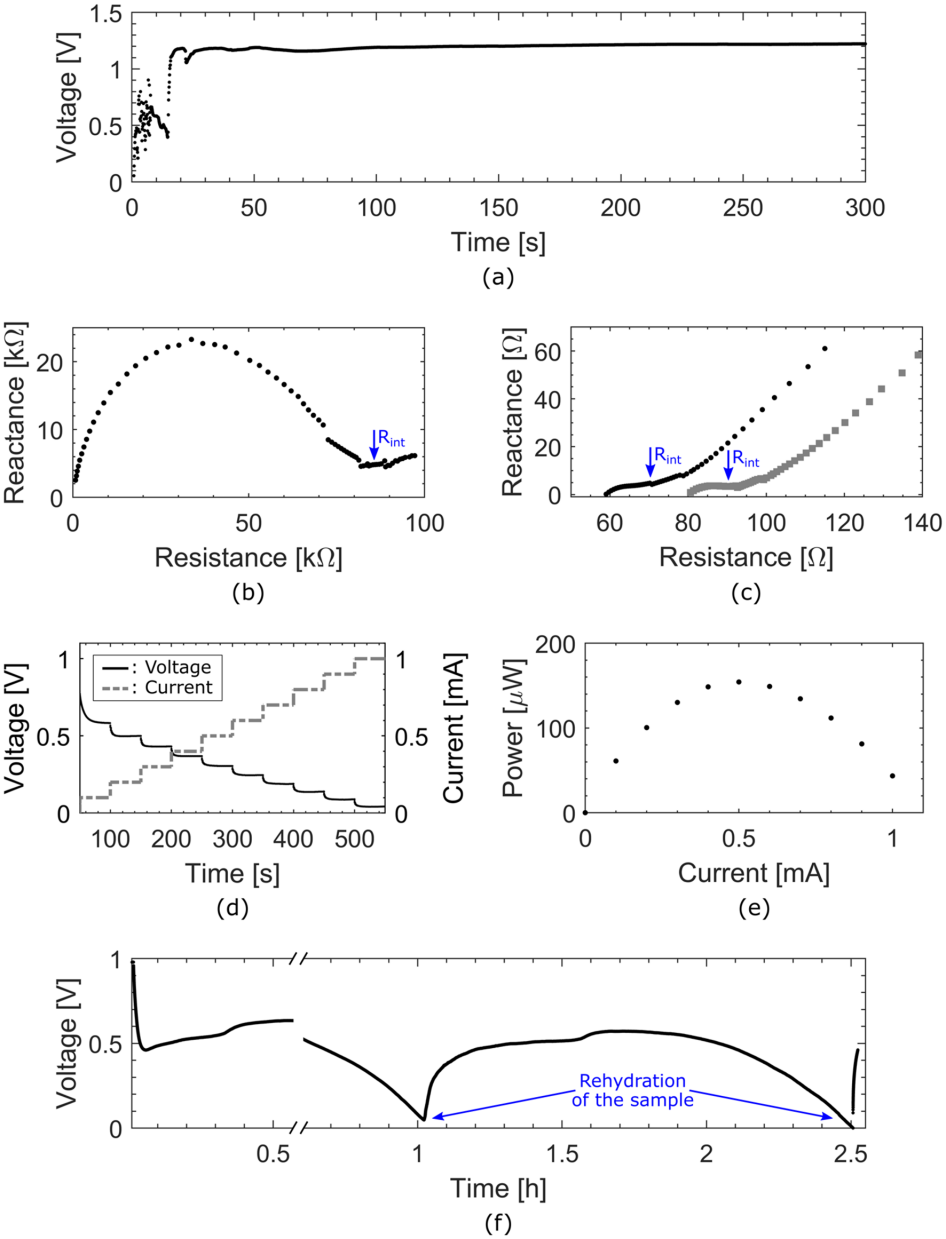
(**a**) Graph of the open circuit potential (OCP) of the single-cell battery as a function of time upon activation. Time zero corresponds to the moment water was dispensed on the activation wick. The battery shows a stable 1.2 V OCP and a 20 s activation time. (**b**) Nyquist plot of the battery before activation, showing an internal resistance R_int_ of 85 kΩ. (**c**) Nyquist plots of the battery immediately upon activation (grey squares) and after 1 h of discharge at 100 μA (black dots), showing internal resistances R_int_ of around 70 Ω and 90 Ω, respectively. (**d**) Chronopotentiogram of the battery (black solid line) and the corresponding current ramp (grey dotted line) as a function of time. (**e**) Graph of the power generated by the battery as a function of the current, showing a maximum of 150 μW at 0.5 mA. (**f**) Chronopotentiogram of the battery at a constant current of 100 μA, showing the process of drying and reactivating the device. The discontinuity in the data points is due to complementary analysis that were carried out on the sample at its peak operating voltage.

The open circuit potential of the battery was measured as a function of time. As presented in Fig. 3a, after water is provided to the system at time zero, it takes less than 20 s for the battery to activate and reach a stable 1.2 V open circuit potential. Electrochemical impedance spectroscopy measurements were also performed on the device before and after activation. The corresponding Nyquist plots are presented in Fig. 3b and c, respectively. The internal resistance decreases by more than three orders of magnitudes, dropping from 85 kΩ to 90 Ω upon activation. The same measurement was performed after discharging the device for 1 h at a constant current of 100 μA. Results presented in Fig. 3c show that the internal resistance further decreased to 70 Ω.

The power capability of the battery was characterized by measuring the operating voltage at different discharge currents ranging from 0.1 mA to 1 mA. Figure 3d shows the measured operating voltage (black solid line) and the corresponding discharge current (grey dotted line) as a function of time. Each current step lasts for 50 s, enough for the operating voltage to reach a stable value. Figure 3e shows the corresponding calculated electrical power as a function of the driving current. The device provided a maximum of 150 μW at 0.5 mA. Higher operating voltage can be achieved by connecting multiple electrochemical cells in series. As shown in Fig. 1c, our two cell battery was capable of powering an alarm clock and its liquid crystal display.

The discharge behavior of the battery was characterized by measuring the operating voltage over an extended period of time at constant current. Figure 3f presents the chronopotentiogram obtained at a constant current of 100 μA. The discontinuity at 30 min in the data points is where the experiment was stopped to measure the power capability of the device close to its peak operating performance. After 1 h of discharge (and the additional power capability measurements), performance significantly decreases due to drying of the paper substrate. At activation, the system was only provided with enough water to saturate the paper substrate which represents approximately 100 mg of water, corresponding to two drops of water dispensed directly on the battery’s wick using a pipette. Upon rehydration with the same amount of water, the battery readily recovers its performance and maintain a stable operating voltage of 0.5 V for more than 1 h. The operating time before rehydration is essentially limited by the amount of water that the paper membrane can absorb. An approach to extending operation time would be to use a larger or thicker wick, effectively using it as a reservoir.

To put these results into perspective it can be useful to look at the requirements of internet of things (IoT) devices. An IoT ecosystem is typically composed of distributed low-power transducers that can communicate with a centralized high-power processing unit. Power requirements varies by several order of magnitudes across the complete ecosystem, from 10 nW to 100 W^24^. As reported by Mish et al., miniaturized FM receivers can operate at around 1 mW. This is slightly above the demonstrated performance of our 1 cm^2^ single cell battery, but still within reach by connecting cells in parallel or by increasing the surface area of the battery. For device operating within the demonstrated performance range, we find hearing aid at 100 μW, RFID tag at 10 mW, electronic watch calculator at 1 μW and quartz oscillator at 100 nW. It should also be noted that low-power electronic devices can remain in standby while consuming as little as 10 nW, significantly decreasing the average power consumption.

## Conclusion

We developed a water-activated paper battery for single-use electronics with low environmental impact. We fabricated single and two cell batteries using stencil printing, and demonstrated that the device can be made to arbitrary shapes and sizes. The electrodes and current collector inks that we developed exhibit shear thinning gel properties, ideal for additive manufacturing techniques like stencil printing and extrusion-based 3D printing. We demonstrated that the battery activates within 20 s and only requires a small amount of water. This activation feature enables on-demand power delivery that can be actively triggered by the user, or passively triggered by the environment. The battery provides an open circuit potential of 1.2 V and achieved a peak power density of 150 μW/cm^2^ at 0.5 mA. As a proof of concept, the two cells battery was used to power an alarm clock. This demonstration shows that despite its limited power density when compared to standard technologies, our battery is still relevant for wide range of low-power electronics and the IoT ecosystem. This work advances the field of disposable electronics and presents a battery technology that balances environmental impact and performance. Future work will investigate the use of green catalysts to improve the oxygen reduction reaction rate, as well as organic anode materials as a replacement for zinc. Life cycle assessment will also be completed to evaluate and compare the environmental impact (CO_2_/kWh) of our paper battery.

## Supplementary Information

Supplementary Information.

## Data Availability

The datasets used and/or analysed during the current study available from the corresponding author on reasonable request.
