# Supplementary material for: Water activated disposable paper battery
Source: Sci Rep. 2022 Jul 28;12:11919. doi: 10.1038/s41598-022-15900-5 (PMC9334575; doi:10.1038/s41598-022-15900-5)
Supplement: Supplementary file 1 — Supplementary Information. [file 41598_2022_15900_MOESM1_ESM.docx]

**Supplementary information**

**
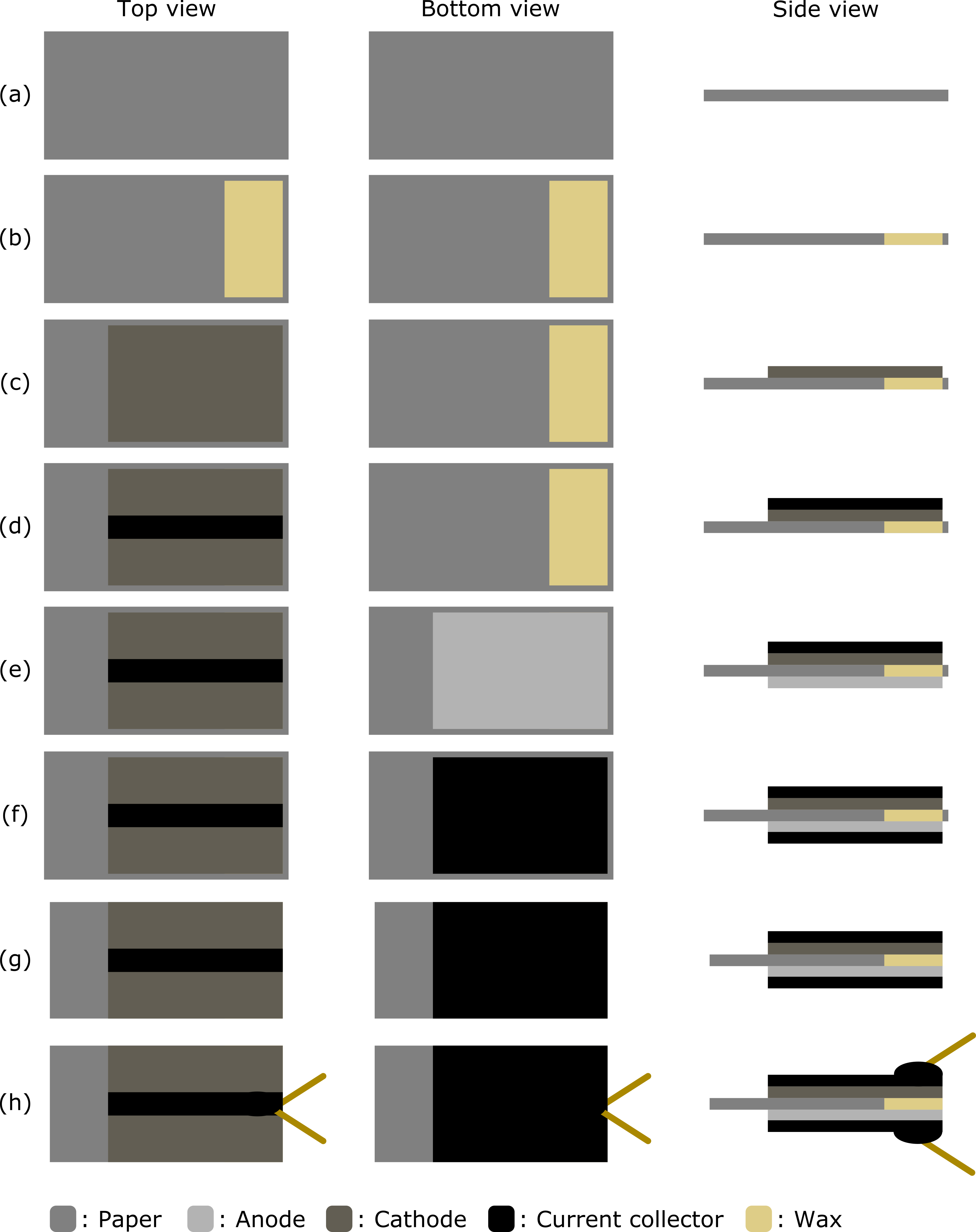
**

**Figure S1:** Illustration of the single-cell battery fabrication process showing top, bottom and side views of the device at every step of the fabrication. (a) The process starts with a paper substrate cut to a slightly larger size than the final desired battery. (b) The paper substrate is impregnated with carnauba wax on one end, immersed in a 3 M aqueous solution of NaCl, and dried. (c) The cathode is stencil-printed and dried in the oven at 60 ^o^C for 10 min. The same printing technique and drying conditions are used to subsequently pattern the (d) top current collector, (e) anode and (f) bottom current collector. (g) The resulting sample is cut to its final shape and (h) lead wires are connected to the battery terminals using the current collector ink.

**
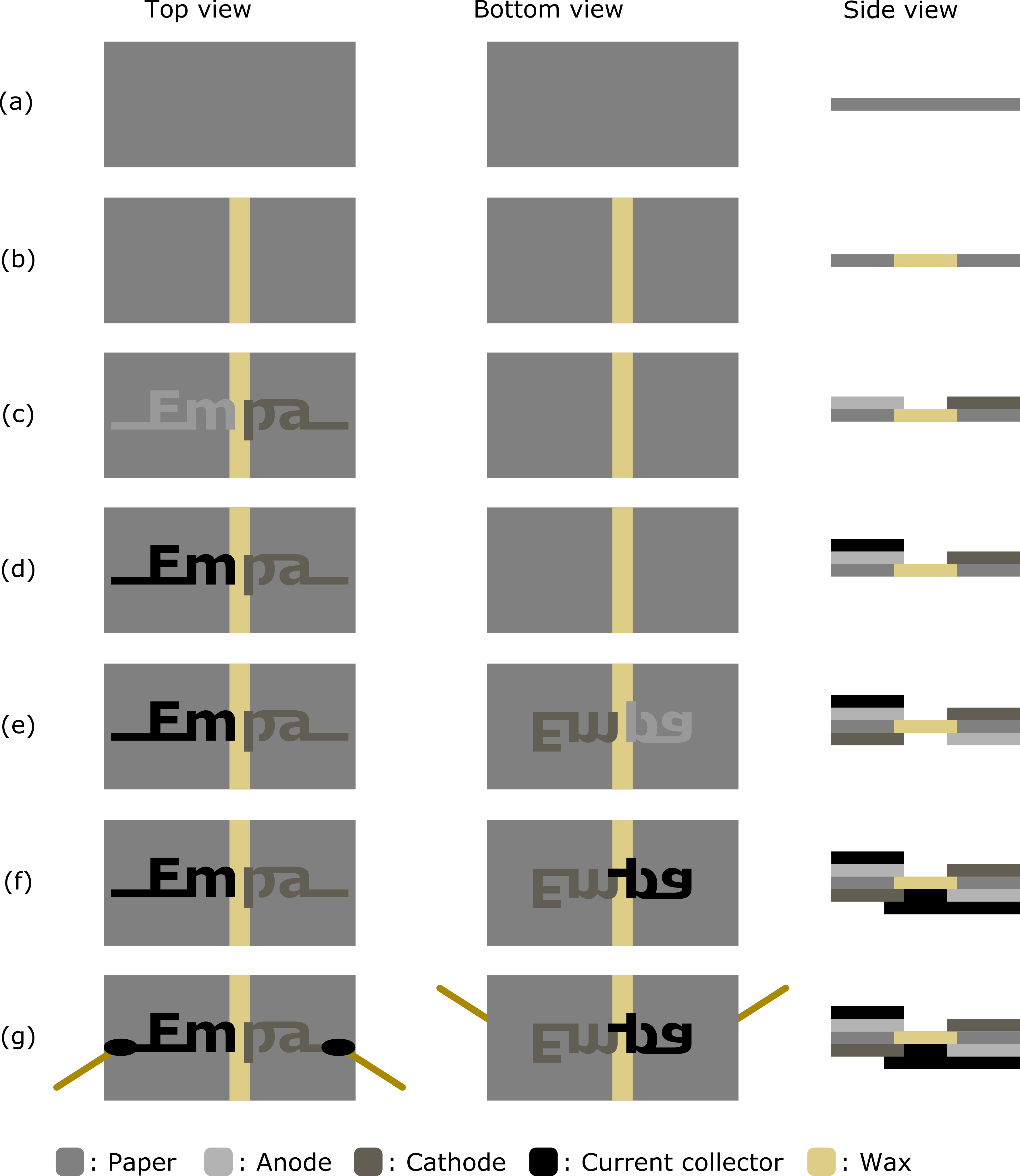
**

**Figure S2:** Illustration of the Empa battery fabrication process showing top, bottom and side views of the device at every step of the fabrication. The side view is limited to the central area, at the border between the two electrochemical cells composing the battery. (a) The process starts with a paper substrate cut to a slightly larger size than the battery design. (b) A stripe of carnauba wax oleogel is stencil-printed on the substrate to isolate the two sides that will become two independent electrochemical cells. The sample is placed on a 100 ^o^C hotplate for a few seconds to let the wax melt and infuse the paper, immersed in a 3 M solution of NaCl, and dried. (c) A cathode and an anode, shaped to spell the name of our research institution (Empa), are stencil-printed on top of the device and dried in the oven at 60 ^o^C for 10 min. The same printing technique and drying conditions are used to subsequently pattern the (d) top current collector, (e) anode and cathode on the bottom side of the substrate, and (f) the bottom current collector. (g) Lead wires are connected to the battery terminals using the current collector ink. The two cells are connected in series. The left and right leads are connected to the anode and cathode of the battery, respectively.
